# Exploration of the lower threshold of iodine intake in Southern Chinese young adults based on ‘overflow theory’ in an iodine balance study

**DOI:** 10.1186/s12937-022-00775-z

**Published:** 2022-05-30

**Authors:** Jun Wang, Hongmin Zhang, Deqian Mao, Hongxing Tan, Wei Yu, Jian Xu, Wenxu Hong, Jianhua Piao, Lichen Yang, Xiaobing Liu, Jiaxi Lu, Weidong Li, Yajie Li, Xiaoli Liu, Xiaoguang Yang

**Affiliations:** 1 Shenzhen Center for Chronic Disease Control, 2021 Bu Xin Road, Luo Hu District, Shenzhen, 518020 Guangdong China; 2 Shenzhen Polytechnic, 7098 Liuxian Avenue, Nanshan District, Shenzhen, 518055 Guangdong China; 3 The Key Laboratory of Micronutrients Nutrition, National Health Commission, National Institute for Nutrition and Health, Chinese Center for Disease Control and Prevention, 29 Nan Wei Road, Xi Cheng District, Beijing, 100050 China

**Keywords:** Iodine, Iodine overflow, Chinese adults, Balance experiment, Euthyroid

## Abstract

**Background:**

Appropriate iodine intake for adults is essential to reduce the prevalence of thyroid diseases, but there is little research data on iodine requirement of Chinese population. This study aimed to explore the iodine requirement of young adults to maintain a healthy status based on ‘overflow theory’.

**Methods:**

Iodine-balance experiment has been performed in this project. We conducted an 18-day study consisted of a 6-day acclimation period and 3 consecutive experimental stages in 37 Chinese healthy young adults (23 female and 14 male). Each stage was consumed for 4 days. Strictly-controlled low-iodine intake diets were provided for adults in the first period, an egg or 125mL milk was added in the second and third period, respectively. The dietary samples, 24-h urine specimens and faeces of volunteers were collected daily for assessment of iodine intake and excretion in volunteers.

**Results:**

Mean values of iodine intake (22.7±3.6, 35.1±3.7, and 52.2±3.8μg/d), excretion (64.7±13.9, 62.3±12.6, and 94.3±14.5μg/d) and iodine balance (-35.2±19.5, -21.0±19.8, and -33.5±26.9μg/d) were significantly different among three periods for male (*P*<0.001 for all); mean values of iodine intake (16.6±3.1, 29.7±2.7, and 48.0±2.7μg/d), and excretion (47.0±9.9, 55.5±8.1, and 75.7±12.4μg/d) were significantly different among three periods for female (*P* < 0.001 for all). No significant difference was observed among the 3 periods for female in the iodine balance (-30.5±9.3, -25.9±7.3, and -27.6±12.1μg/d). The linear regression equation of iodine excretion on iodine intake was Y=0.979X+37.04 (male) and Y=0.895X+31.48 (female). Compared with stage 2, iodine excretion increments in stage 3 had exceeded the iodine intake increment for men. The ratio of increment was 1.675 for male when the average iodine intake was 52.2μg/d in stage 3. When the iodine excretion increment equaled to the iodine intake increment, the daily iodine intake of men was 47.0μg.

**Conclusion:**

We have evaluated the iodine requirement of young adults in southern China based on overflow theory. Our results indicate the lower limit of iodine requirement for Chinese young men is 47.0μg/d. The trial was registered at www.chictr.org.cn as ChiCTR1800014877.

**Supplementary Information:**

The online version contains supplementary material available at 10.1186/s12937-022-00775-z.

## Background

Iodine is one of the essential trace elements for the human body. It plays an important role in the synthesis of thyroid hormone and has been regarded as a determinant of thyroid disorders [1]. Long-term iodine insufficiency intake is a risk factor for nodules, subclinical hypothyroidism and thyroid goitre [2, 3]. Since universal salt iodization (USI) was implemented in 1995, China has made remarkable achievements in the prevention and control of iodine deficiency disorders (IDD). By the end of 2015, 94.2% of rural China had reached the target of eliminating IDD) [4]. A report from China National Expert Committee on Food Safety Risk Assessment showed that the iodine nutritional status of Chinese residents is generally at an appropriate and safe level in 2010 [5]. However, several recent studies reported that excessive iodine intake may associated with high thyroid volume for children [6, 7] and hypothyroidism, thyroid nodules for adults [8, 9]. Furthermore, along with the increasing of thyroid cancer, the relationship between iodine excess and USI has caused more concern [10]. Considering these factors, China revised the iodized salt standard from 35 mg/kg to 20~30 mg/kg in 2012, resulting average iodine content in edible salt decreased from 30.7 mg/kg in 2005 to 24.3 mg/kg in 2014 [11]. The new iodized salt criterion inevitably has an impact on the total dietary iodine intake of Chinese. Therefore, a reasonable iodine dietary reference intake should be established to ensure public health.

There are obvious regional differences in dietary iodine reference intake throughout the world. The United States and Canada set the iodine estimated average requirement (EAR) of 95 μg/d for adults using data published in the 1960s on thyroid iodine metabolism, turnover and balance studies [12–14]. Because of the lack of experimental data on Chinese population, Chinese Nutrition Society determined the EAR for iodine as 85μg/d considering the difference in body weight between adult Chinese population and Western population [15]. In addition, with the implementation of USI, the iodine nutritional status of the populations in the world has undergone tremendous changes, and thus thyroid iodine content and turnover in euthyroid subjects recently are different from that in the middle of last century. Therefore, the iodine requirement based on the results of previous studies has been difficult to adapt to the change of iodine requirement under the new global iodine nutrition background.

Metabolic balance study is a classical method to determine nutrient requirements by assessing the relationship between total nutrient intake and excretion [16]. For iodine-sufficient adults, the iodine balance should be zero balance for a while, that is, total iodine intake and excretion are basically equal. Under normal conditions, about 90% of the iodine in the human body is excreted through the urine, and about 10% of the iodine is excreted through the feces, then the iodine excretion through the urine and feces can represent the total iodine excretion [4]. However, there are very few iodine balance studies have been reported in China due to the difficulty in controlling dietary iodine intake and the difficulty in collecting complete 24 hour urine over a period of time [17].

Based on the balance study theory and iodine nutritional status of Chinese adults, the ‘iodine overflow’ theory was proposed in 2020 and had been applied to explore the iodine requirement of the northern population [18], that is: 1) healthy adults can absolutely and effectively utilize iodine, and when the iodine intake is sufficient for the body's metabolic needs and necessary storage, the excess iodine will be excreted; 2) iodine excretion increases with the increase of iodine intake and when iodine intake exceeds a certain threshold, the increased dietary iodine intake will be completely excreted, this threshold should be the lower limit of iodine dietary reference intake for adults. As China has vast territory, there are great differences in dietary habits and food iodine content between south and north. The aim of present study was to determine lower threshold of iodine intake for young adults in South China according to the ‘iodine overflow’ theory.

## Methods

### Subjects

We recruited 39 volunteers aged 18~23 years old from Shenzhen Polytechnic in 2018. The weight and height of the volunteers were measured by calibrated instruments to calculate body mass index (BMI). All the subjects were given unified physical examinations in professional hospital before and after dietary interventions. Overnight (≥8 hour) fasting blood specimens were collected from each participant for measurement of thyroid hormones, inflammatory, liver and kidney function indicators. Volunteers were included in our study if they fulfilled the following criteria: 1) normal thyroid function (defined as thyroid-stimulating hormone (TSH) of 0.35-5.5 mIU/l, free triiodothyronine (FT3) of 3.5-6.5 pmol/l and free thyroxine (FT4) of 11.5-22.7 pmol/l), no thyroid disorders and treatment history; 2) no exposure to iodine-containing dietary supplement or iodine-containing medication; 3) normal urinary iodine concentration; 4) no functional constipation or diarrhea.

This trial was registered in the Chinese Clinical Trial Registry (ChiCTR1800014877). All procedures involving human participants were approved by the Ethical Committee of the Chinese Center for Disease Control and Prevention. All subjects provided their written informed consent prior to participation.

### Sample size

The sample sizes were calculated by using Gpower 3.1 software. As the study was a before–after self-controlled study, it was estimated that 34 participants would be required for a paired t-test to detect a medium effect size (*F*=0.5) with a statistical power (1–β error probability) of 80% and an α of 0.05. Thus, our sample size of 37 was adequate.

### Study design

**Fig. 1:**
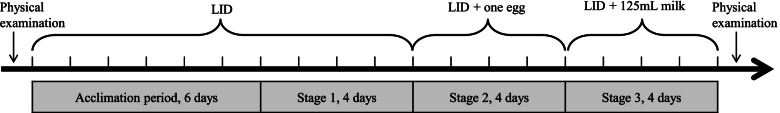
Study design flow chart for 37 Chinese young adults

Considering the potential effect of female physiological period, the whole study was executed limited to a total duration of 18 days, which was divided into acclimation period and three low-iodine intake stages. The project took place in late spring and early summer (March and April) in the southern Chinese metropolis of Shenzhen, with ambient temperatures around 20 degrees. The subjects were required to avoid intensive physical activities during the whole study for controlling iodine excretion from sweat. Diet plans were carefully designed based on balanced meals avoiding from iodine-rich foods and condiments (i.e., kelp, seaweed, iodized salt, etc.). Serving foods and drinking water were restrictively monitored and provide to all the participants. Acclimation period was 6 days before the beginning of the studies in order to reducing the effect of stored iodine in the thyroid (Fig. 1). Subsequently, there were three low-iodine diet (LID) stages, each lasting 4 days (12 days total). In stage 2 and 3, a hard-boiled egg (approx. 50g each) or 125 mL of milk was used for intervention, respectively. The dietary samples, 24-h urine specimens and faeces of volunteers were collected daily by our investigators.

### Diet design, preparation and processing

According to the basic data of the typical dietary pattern obtained from the school canteen and market supply, a 12-day recipe met the principles of nutrient balance was designed by nutritionists. Daily recipe contained staple food, vegetables, fruits, beans and meat, but seaweed and kelp were prohibited. Before food processing, the iodine contents of all cooked foods, including raw materials, compound condiments, snacks and drinking water, were carefully determined. Diets contained different levels of iodine in different periods. All diet cooked with non-iodized salt by the canteen staff. Six drinking water samples collected in school have been detected, and the mean water iodine was 11.8 μg/L. To avoid the bias of iodine content in different drinks, commercially available pure water in the supermarket was used to cook food and drink for subjects. The 12-day recipe used in this study was shown in Supplemental Table 1.

### Sample collection

Participants were provided with a plastic beaker (1L) and two plastic bags with sealed lid (5L), and they were guided and demonstrated how to collect the 24-h urine by staff. The 24-h urine was collected for 12 consecutive days from 7 am on the first day to 7 am on the 13th day of the trial. Upon completion of the 24-h collection, the urine volume of each participant was measured and four aliquots were frozen at -20°C for later analysis.

Carmine capsule was administered to all subjects before breakfast to mark feces on the first day, fifth day, ninth day and the thirteenth day respectively. Fecal specimens of each subject were collected according to the labeled red pigmentation at each stage. Once the feces of each stage were completely collected, they were weighed, homogenized, proportionally taken a portion for about 200g and pressed using moulding followed by lyophilization and freezing at -20°C.

During the study, all the diet offered to the subjects was cooked by the university canteen according to the recipe. Trained staff weighed the food supplied for the subjects before and after each meal to access actual intake. A duplicate dietary sample for each meal was collected and homogenized in one hour, and then stored in the refrigerator at -20°C before detection.

### Laboratory analysis

Quantitative rapid test kits (Conson Biochemicals, China) were used to detect 24-hour urinary iodine concentration every day to prevent iodine intake other than the diet we provided. Iodine content in food [19], urine [20] and faeces [19] were determined using arsenic-cerium catalytic spectrophotometry at National Iodine Deficiency Disease Reference Laboratory in Beijing. The serum thyrotropin (TSH), free thyroxin (FT4) and free triiodothyronine (FT3) were measured in all participants by an automated chemiluminescence immunoassay analyzer (Bayer ADVIA Cetaur System, Bayer Healthcare, Germany).

### Quality control

Multiple quality control (QC) measures were introduced to guarantee the reliability of the study findings. Prior to the beginning of the survey, all the investigators were trained uniformly for recording weighed food by the principal investigator from National Institute for Nutrition and Health of Chinese CDC. Double entry data have been used to ensure accuracy and completeness. One of the difficulties and limitations in the study was the 24-hour urine samples collection. The total urine volume and 24-hour urinary creatinine excretion were applied to validate the completeness of the 24-hour collection sample. Eating out or consuming food not provided by the project team, was prohibited. The iodine concentrations of 24-hour urine samples were determined promptly every day, and 24-hour urine iodine excretion was calculated to monitor volunteers' dietary iodine intake. Carmine red, a biologically inert substance that was rapidly excreted in faeces, was administered to all participants at the beginnings and ends of each stage to distinguish different stages of stool samples.

### Statistical analysis

Data were analyzed using Excel and IBM SPSS 21.0 Statistics software. The normal distribution of data was checked using the Shapiro-Wilk test. Normally distributed data were expressed as mean ± SD, while non-normally distributed data were expressed as the median (25th-75th percentiles). Differences among the 3 periods were analyzed by one-way repeated measures ANOVA and by the use of Bonferroni correction for multiple comparisons. A *P* value < 0.05 was considered as statistically significant.

### ‘Iodine overflow’ hypothesis

‘Iodine overflow’ hypothesis [18] was first proposed by our group in 2020, which was appropriate for exploring the recommended nutrient intake of iodine under saturation state. Shenzhen, as a coastal city, has implemented USI policy over twenty years and the iodine nutrition status of its residents is appropriate. Therefore, dietary iodine was adequate to meet needs for thyroid hormone synthesis and required storage, while the remaining iodine maybe excreted by ‘overflow’. As we know, iodine excretion increases with iodine intake in certain extent. Dietary contained 3 concentrations of iodine was successively provided for all subjects in 3 periods. Data of iodine excretion increment and intake increment were obtained using the change of iodine intake and excretion between different periods. Ratio was calculated by the iodine excretion increment to iodine intake increment. A scatter diagram was plotted by ratio vs iodine intake, and then the iodine intake was calculated when ratio equaled to 1 with linear regression method.

## Results

### Characteristic of study population

Of the 39 young healthy adults recruited, 37 (23 female and 14 male) successfully completed this study were included. As shown in Table 1, the means of average age and BMI for all 37 participants were 20.1±1.0 years and 20.5±2.8 kg/m^2^, respectively. Median urinary iodine concentration (UIC) at screening was 121.5 (90.9-181.8) μg/L and 126.4 (73.9-161.9) μg/L for male and female, respectively. After acclimation period, the UIC was 31.5 (25.6-43.3) μg/L for male and 20.0 (15.2-31.7) μg/L for female.

**Table 1:** General characteristics of the subjects

	All (*n*=37)	Male (*n*=14)	Female (*n*=23)
Age^a^, year	20.1±1.0	20.2±0.7	20.1±1.1
Height^a^, cm	165.2±9.1	174.5±6.1*	159.5±4.9
Weight^a^, kg	56.6±13.2	68.2±13.9*	49.6±6.4
BMI^a^, kg/m^2^	20.5±2.8	22.3±3.4*	19.4±1.7
UIC^1, b^, μg/L	126.4 (76.2-165.2)	121.5 (90.9-181.8)	126.4 (73.9-161.9)
UIC^2, b^, μg/L	25.3 (17.7-33.6)	31.5 (25.6-43.3)	20.0 (15.2-31.7)

^a^Values are means ± SDs; ^b^Values are medians (P25-P75); *BMI* Body mass index, *UIC*^1^ urinary iodine concentration at screening, *UIC*^2^ urinary iodine concentration after acclimation period; *P<0.05

The level of TSH, FT4 and FT3 were all within the normal reference value (Table 2). The measured mean ± SD serum TSH, FT4 and FT3 of male before trial (1.98±0.72 μIU/ml, 19.62±2.48 pmol/L and 5.67±0.45 pmol/L) had no significant difference compared with the values from after trial (2.02±0.84 μIU/ml, 20.54±2.23 pmol/L and 5.61±0.53 pmol/L, *P* > 0.05). Similar results were seen in female.

**Table 2:** Comparison of thyroid function parameters before and after trial (mean ± SD)

	FT4 (pmol/L)	FT3 (pmol/L)	TSH (μIU/ml)
Male (*n*=14)			
Before trial	19.62±2.48	5.67±0.45	1.98±0.72
After trial	20.54±2.23	5.61±0.53	2.02±0.84
*P* value	0.167	0.744	0.904
Female (*n*=23)			
Before trial	17.74±2.09	4.92±0.6	1.86±0.91
After trial	17.44±2.15	4.92±0.59	1.72±0.94
*P* value	0.330	0.970	0.079
All (*n*=37)			
Before trial	18.45±2.4	5.2±0.65	1.91±0.83
After trial	18.61±2.64	5.18±0.65	1.83±0.89
*P* value	0.604	0.797	0.147

*FT*4 free thyroxine, *FT*3 free triiodothyronine, *TSH* thyroid stimulating hormone

### Dietary iodine intake, urinary and fecal iodine excretion

The values of iodine intake, iodine excretion and iodine balance of 37 volunteers at different iodine doses are shown in Table 3. A total of 359 samples were analyzed in this study, while others were excluded because their urine collection were not complete. Significant differences in total iodine intake were found among all 3 doses for both males and females (*P*<0.001 for all comparisons). Among different iodine intake statuses, iodine excretion differed in both of the genders (*P* < 0.001). There were no significant differences in iodine retention among the 3 periods for female (*P* = 0.262).

**Table 3:** Summary of daily iodine intake, iodine excretion, and iodine retention for the subjects (mean ± SD)

	Stage 1	Stage 2	Stage 3	*P*^*c*^
Male (*n*=14)				
Total iodine intake, μg/d	22.7±3.6	35.1±3.7^a^	52.2±3.8^ab^	<0.001
Urine iodine, μg/d	56.4±13.2	51.4±11.5 ^a^	78.9±15.4 ^ab^	<0.001
Feces iodine, μg/d	9.1±4.0	11.9±3.4	16.7±5.8^a^	<0.001
Total iodine excretion, μg/d	64.7±13.9	62.3±12.6	94.3±14.5^ab^	<0.001
Iodine balance, μg/d	-35.2±19.5	-21.0±19.8^a^	-33.5±26.9^b^	<0.001
Female (*n*=23)				
Total iodine intake, μg/d	16.6±3.1	29.7±2.7 ^a^	48.0±2.7 ^ab^	<0.001
Urine iodine, μg/d	40.8±9.5	47.8±8.1^a^	66.4±11.0^ab^	<0.001
Feces iodine, μg/d	6.2±1.6	8.0±3.1	9.7±6.5^a^	0.044
Total iodine excretion, μg/d	47.0±9.9	55.5±8.1^a^	75.7±12.4^ab^	<0.001
Iodine balance, μg/d	-30.5±9.3	-25.9±7.3	-27.6±12.1	0.262
Total (*n*=37)				
Total iodine intake, μg/d	18.9±4.4	31.7±4.1 ^a^	49.6±3.7 ^ab^	<0.001
Urine iodine, μg/d	46.7±13.3	49.2±9.6	71.1±14.0^ab^	<0.001
Feces iodine, μg/d	7.3±3.1	9.5±3.7^a^	12.3±7.1^a^	<0.001
Total iodine excretion, μg/d	53.7±14.3	58.1±10.4 ^a^	82.7±15.9 ^ab^	<0.001
Iodine balance, μg/d	-32.3±14.0	-24.1±13.4^a^	-29.8±18.9	0.003

^a^*P* < 0.05 between each periods with the stage 1; ^b^*P* < 0.05 between each stages with the stage 2; ^c^the differences among the three stages were evaluated using repeated-measure ANOVA with Bonferroni correction

**Fig. 2:**
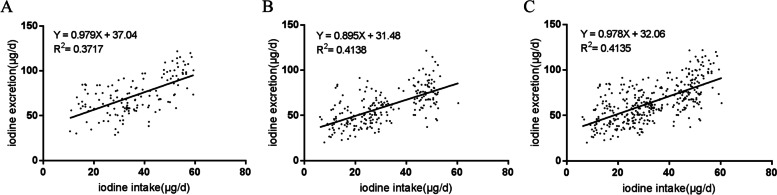
Regression of daily iodine excretion on iodine intake. Each point represent 1 day data of each subject. **A** Male, data included 139 samples from 14 young male; **B** Female, data included 220 samples from 23 young female; **C** Total subjects, data included 359 samples from 37 young adults

Figure 2 displayed the relationship between iodine excretion and iodine intake for male (Fig. 2A), female (Fig. 2B) and all subjects (Fig. 2C), respectively. The regression equations were Y=0.979X+37.04 (male) and Y=0.895X+31.48(female). The intercepts 37.04μg/d and 31.48μg/d represented an indispensable iodine excretion, which were equally different from zero (*P*<0.001).

Iodine excretion increment increased with increasing iodine intake increment as shown in Table 4. Compared with stage 1, the average daily iodine intake for male increased 12.4μg in stage 2, while the average daily iodine excretion decreased 3.2μg, and the ratio was -0.324. Furthermore, the average iodine intake in stage 3 was 17.7μg/d more than stage 2, while the average iodine excretion increment was 29.3μg/d. The ratio of increment was 1.675 for male when the average iodine intake was 52.2μg/d in stage 3. It represented that the increment of iodine intake was all excreted.

**Table 4:** Changes in the ratio of iodine intake to excretion increment (μg/d)

Variables	period2(△increment)			period3(△increment)		
	△increment_in_^a^	△increment_ex_^b^	ratio^c^	△increment_in_^a^	△increment_ex_^b^	ratio^c^
Male	12.4	-3.2	-0.324	17.7	29.3	1.675
Female	13.0	9.2	0.699	18.5	19.0	1.034
Total	12.8	4.3	0.293	18.2	22.9	1.276

^a^△increment_in_ was calculated using the iodine intake of latter stage minus the previous stage; △increment_ex_ was calculated using the iodine excretion of latter stage minus the stage period ; ratio = △increment_ex_/△increment_in_

**Fig. 3:**
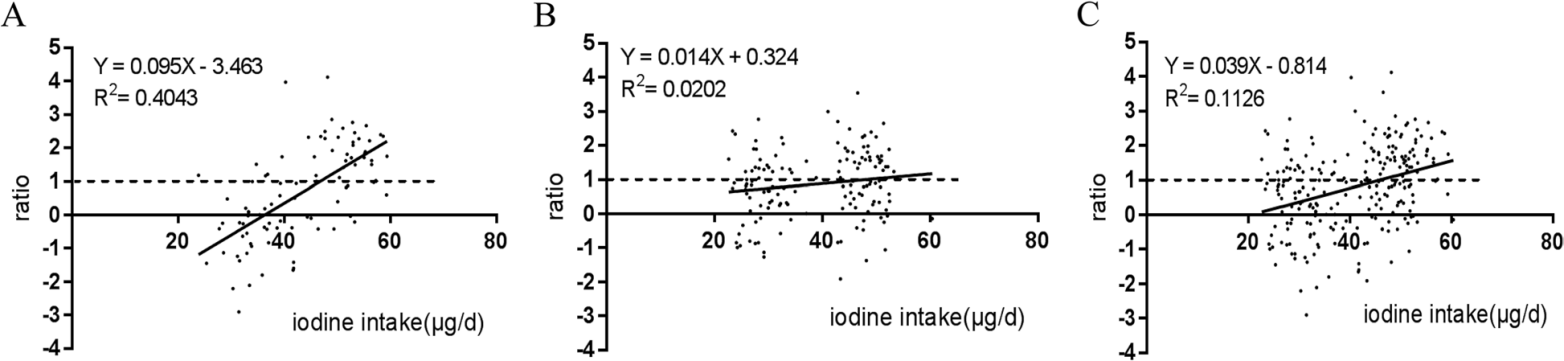
Regression of ratio on iodine intake. ratio=△increment_ex_(a)/△increment_in_(b). (a)△increment_ex_: iodine excretion increment, (b) △increment_in_: iodine intake increment. **A** Male, data included 91 samples from 14 young male ;**B** Female, data included 144 samples from 23 young female; **C** Total subjects, data included 235 samples from 37 young adults

There was a significantly positive correlation between iodine increment ratio (ratio=△increment_ex_/△increment_in_) and iodine intake for male (*r*=0.64, *P*<0.001), but not for female (*r*=0.14, *P*=0.09). Figure 3 showed linear relationship between iodine increment ratio and iodine intake, the equations of the least-squares line were Y=0.095X-3.463 for male, and Y=0.014X+0.324 for female. The intercept (-3.463) and the slope (0.095) of male were significantly different from zero (*p*< 0.001). When the ratio was equal to 1, that was, the increment in daily iodine excretion was equal to the increment in daily iodine intake, the minimum iodine requirement of male was achieved at an iodine intake of 47.0μg/d.

## Discussion

Mean iodine intake of all subjects in 3 periods was 18.9±4.4, 31.7±4.1 and 49.6±3.7μg/d, respectively. The results showed that iodine intake of young adults was consistently lower than 60μg/d in three periods, which was considerably lower than 120μg/d recommended by Chinese Nutrition Society [15]. As almost all subjects were in negative iodine balance during the study, and balance study cannot define iodine requirement, we use ‘iodine overflow’ method to evaluate the lower threshold of iodine requirement of young adults. We observed that the ratio of iodine excretion increment and iodine intake increment increasing with the iodine intake. When the ratio equaled one, value of iodine intake 47.0μg/d was considered as the lower threshold of daily iodine intake in Chinese young male adults.

In our opinion, the negative iodine balance might be related to the iodine status of the subjects who were in an ‘over-saturated state’. First of all, Shenzhen as a coastal city located in southern China has been reached the standard of eliminating IDD many years ago. Andersson [21] considered that the iodine stored in the thyroid gland of population is sufficient to ensure adequate hormone synthesis and secretion where USI has been effective for at least 2 years. Secondly, the 37 young adults who lived in Shenzhen at least 2 years were in an adequate nutritional iodine status before entering our study with UIC 126.4μg/L defined by the WHO standard [22]. We also observed urine iodine excretion increased with iodine intake although UIC was below 100μg/L (iodine deficiency according to WHO) during the study. However, a recent study suggested that Chinese pregnant women with UIC of 107.4μg/L (less than the WHO’s 150μg/L criterion) maintained thyroid function in both themselves and their newborn babies [23]. Last but not least, thyroid function was detected to evaluate iodine status of subjects with low intakes (from 18.9 to 49.6μg/d) in our study. TSH, FT4 and FT3 were not only within the normal range but also had no difference before and after this study.

The balance study was usually used to determine iodine EAR of population by assessing the total iodine intake and excretion [17, 24]. From the perspective of balance study, the results of our study were consistent with previously reported findings of negative iodine balance in healthy subjects [25–27]. Malvaux [25] found the healthy children and adolescents were in negative balance on low iodine intake of 32μg/d, similar to the iodine intake in stage 2 of our study (31.6 μg/d). One may argue that the low iodine intake lead to the negative iodine balance. A balance study conducted by Vought [28] demonstrated that all of the healthy euthyroid volunteers were in negative balance even though they were fed with 3 levels of iodine intake ranging from 65.8 to 450.4 μg/d in random order. Nevertheless, it is impossible for healthy adults to maintain ‘negative iodine balance’ constantly, and the classical iodine balance experiment cannot reasonably explain this phenomenon.

Considering that the EAR cannot be calculated from our data, the method of iodine overflow was used to determine the lower limit of iodine requirement for young adults. It does not require the population to reach zero iodine balance, which to some extent makes up for the defect that the iodine balance method cannot evaluate the iodine requirement in the case of negative iodine balance. DeGroot [14] had estimated the thyroid absolute iodide uptake in four normal thyroid function subjects by using radioactive ^131^I. The results showed that the mean of absolute iodine uptake was 54μg/d by the method of Riggs [29], close to lower limit of iodine requirement of men in our study (47.0μg/d). Nevertheless, considering ethical issues, it is currently impossible to conduct human trials using radioisotope labeling methods. Therefore, our ‘iodine overflow’ hypothesis may open up a new way for establishing the dietary reference intake of iodine for healthy adults even if they are in negative iodine balance.

Yang et al. [18] first tried to explore the northern population iodine requirements according to ‘iodine overflow’ theory. They found that daily iodine intake of 63.4 μg might be adequate for males [18], while our results showed that 47.0 μg/d was the minimum iodine requirement of young males. Reasons for this discrepancy might be that the baseline iodine nutritional status and dietary habits among populations throughout north and south China are different [30]. Moreover, our data indicated that females had higher minimum iodine requirement than males which was consistent with Yang et al. [18]. However, further researches are needed to verify the result.

Our present study has several limitations. First, a major limitation of this study is the narrower ranges of iodine exposure (18.9μg/d to 49.6μg/d) compared with previous reports, and thus contributes no information as to the effect of high iodine intakes on iodine balance. However, multiple iodine intake levels were designed at narrow range may improve the accuracy of lower threshold results. Second, it may also be objected that the total iodine excretion is underestimated because iodine losses from sweat, skin and menstruation are not measured. This study was conducted in the spring and volunteers were required to avoid high-intensity physical activity during the test to reduce sweating. Third, in order to avoid the effect of menstruation on iodine excretion and to maintain the compliance of subjects, we avoided the menstrual period and caused a relatively short experimental period. Long term influence of such iodine intake level on thyroid function remains to be determined.

## Conclusions

In conclusion, to the best of our knowledge, this is the first research to determine the iodine requirement for southern Chinese adults based on ‘iodine overflow’ hypothesis. We found that the iodine requirement for healthy Chinese young men was less than current EAR. Further researches are needed to verify our results in a wider ranges of iodine exposure and pay more attention to the iodine status and health of women.

## Supplementary Information

**Additional file 1: Supplemental Table 1.** Dietary recipe with iodine content during the experiment (μg/kg).

## Data Availability

All relevant data are included in this manuscript.

## Abbreviations

USI: Universal salt iodization
EAR: Estimated average requirement
TSH: Serum thyrotropin
FT4: Free thyroxin
FT3: Free triiodothyronine
UIC: Urinary iodine concentration
LID: Low-iodine diet

## References

[1] Zimmermann MB, Boelaert K (2015). Iodine deficiency and thyroid disorders. Lancet Diabetes Endocrinol.

[2] Taylor PN, Albrecht D, Scholz A, Gutierrez-Buey G, Lazarus JH, Dayan CM (2018). Global epidemiology of hyperthyroidism and hypothyroidism. Nat Rev Endocrinol.

[3] Dumont JE, Ermans AM, Maenhaut C, Coppée F, Stanbury JB (1995). Large goitre as a maladaptation to iodine deficiency. Clin Endocrinol (Oxf).

[4] Chinese Society of endemiology/Chinese Nutrition Society/Chinese Society of Endocrinology (2018). Iodine supplementation guidelines for Chinese residents.

[5] China National Expert Committee on Food Safety Risk Assessment (2010). Salt iodization and risk assessment of iodine status in Chinese population.

[6] Zimmermann MB, Ito Y, Hess SY, Fujieda K, Molinari L (2005). High thyroid volume in children with excess dietary iodine intakes. Am J Clin Nutr.

[7] Chen W, Li X, Wu Y, Bian J, Shen J, Jiang W (2017). Associations between iodine intake, thyroid volume, and goiter rate in school-aged Chinese children from areas with high iodine drinking water concentrations. Am J Clin Nutr.

[8] Shan Z, Chen L, Lian X, Liu C, Shi B, Shi L (2016). Iodine Status and Prevalence of Thyroid Disorders After Introduction of Mandatory Universal Salt Iodization for 16 Years in China: A Cross-Sectional Study in 10 Cities. Thyroid.

[9] Laurberg P, Cerqueira C, Ovesen L, Rasmussen LB, Perrild H, Andersen S (2010). lodine intake as a determinant of thyroid disorders in populations. Best Pract Res Clin Endocrinol Metab.

[10] Chen W, Zheng R, Baade PD, Zhang S, Zeng H, Bray F (2016). Cancer statistics in China, 2015. CA Cancer J Clin.

[11] Ministry of Health of the People’s Republic of China (2011). Iodine content in edible salt (GB 26878-2011).

[12] Fisher DA, Oddie TH (1969). Thyroidal radioiodine clearance and thyroid iodine accumulation: Contrast between random daily variation and population data. J Clin Endocrinol Metab.

[13] Fisher DA, Oddie TH (1969). Thyroid iodine content and turnover in euthyroid subjects: Validity of estimation of thyroid iodine accumulation from short-term clearance studies. J Clin Endocrinol Metab.

[14] DeGroot LJ (1966). Kinetic analysis of iodine metabolism. J Clin Endoerinol Metab.

[15] Chinese Nutrition Society (2014). Chinese DRIs handbook.

[16] Mertz W (1987). Use and misuse of balance studies. J Nutr.

[17] Tan L, Tian X, Wang W, Guo X, Sang Z, Li X (2018). Exploration of the appropriate recommended nutrient intake of iodine in healthy Chinese women: an iodine balance experiment. Br J Nutr.

[18] Yang LC, Wang J, Yang JJ, Zhang HD, Liu XB, Mao DQ (2020). An iodine balance study to explore the recommended nutrient intake of iodine in Chinese young adults. British J Nutr.

[19] Ministry of Health of the People’s Republic of China (2008). Method for determination of iodine in foodstuff by As3+-Ce4+ catalytic spectrophotometry (WS 302-2008).

[20] National Health Commission of the People’s Republic of China (2016). Determination of iodine in urine-part 1: As3+-Ce4+ catalytic spectrophotometry (WS/T 107.1-2016).

[21] Secretariat WHO, Andersson M, de Benoist B, Delange F, Zupan J (2007). Prevention and control of iodine deficiency in pregnant and lactating women and in children less than 2-years-old: conclusions and recommendations of the Technical Consultation. Public Health Nutr.

[22] WHO/UNICEF/ICCIDD (2007). Assessment of iodine deficiency disorders and monitoring their elimination: a guide for programme managers, 3.

[23] Zhang H, Wu M, Yang L, Wu J, Hu Y, Han J (2019). Evaluation of median urinary iodine concentration cut-off for defining iodine deficiency in pregnant women after a long term USI in China. Nutr Metab (Lond).

[24] Dold S, Zimmermann MB, Baumgartner J, Davaz T, Galetti V, Braegger C (2016). A dose-response crossover iodine balance study to determine iodine requirements in early infancy. Am J Clin Nutr.

[25] Malvaux P, Beckers C, De Visscher M (1969). Iodine balance studies in nongoitrous children and in adolescents on low iodine intake. J Clin Endocrinol Metab.

[26] Harrison MT, Harden RM, Alexander WD, Wayne E (1965). Iodine Balance Studies in Patients with Normal and Abnormal Thyroid Function. J Clin Endocrinol Metab.

[27] Bakker B, Vulsma T, de Randamie J, Achterhuis AM, Wiedijk B, Oosting H (1999). A negative iodine balance is found in healthy neonates compared with neonates with thyroid agenesis. J Endocrinol.

[28] Vought RL, London WT (1967). Iodine intake, excretion and thyroidal accumulation in healthy subjects. J Clin Endocrinol Metab.

[29] Riggs DS (1952). Quantitative aspects of iodine metabolism in man. Pharmacol Rev.

[30] Sang Z, Wang PP, Yao Z, Shen J, Halfyard B, Tan L (2012). Exploration of the safe upper level of iodine intake in euthyroid Chinese adults: a randomized double-blind trial. Am J Clin Nutr.

