# Supplementary material for: Exploration of the lower threshold of iodine intake in Southern Chinese young adults based on ‘overflow theory’ in an iodine balance study
Source: Nutr J. 2022 May 30;21:35. doi: 10.1186/s12937-022-00775-z (PMC9150309; doi:10.1186/s12937-022-00775-z)
Supplement: Supplementary file 1 — Additional file 1: Supplemental Table 1. Dietary recipe with iodine content during the experiment (μg/kg). [file 12937_2022_775_MOESM1_ESM.docx]

**Supplemental Table 1. Dietary recipe with iodine content during the experiment（μg/kg）**

| Day 1 | Iodine content | Day 2 | Iodine content | Day 3 | Iodine content | Day 4 | Iodine content |
| --- | --- | --- | --- | --- | --- | --- | --- |
| Period 1 | | | | | | |  |
| Oyster sauce fried lettuce | 12.5 | Asparagus lettuce | 0 | Shredded potatoes | 12.5 | Shredded potatoes | 0 |
| Bean paste bun | 0 | Fried cake | 12.2 | Bean paste bun | 0 | Bean paste bun | 17.1 |
| soymilk | 18.1 | Porridge | 0 | soymilk | 18.1 | Porridge | 0 |
| Mustard tuber | 25.5 | Mustard tuber | 25.5 | Mustard tuber | 25.5 | Mustard tuber | 25.5 |
| Rice | 6 | Rice | 6 | Rice | 6 | Rice | 6 |
| Braised pork | 18.2 | Cabbage | 0 | [Chinese kale](http://dict.cnki.net/dict_result.aspx?searchword=%e8%8a%a5%e8%93%9d&tjType=sentence&style=&t=chinese+kale) | 34.4 | Green pepper | 6.9 |
| Dry fried cauliflower | 0 | Shredded potatoes | 14.9 | Parsley fried carrot | 15.4 | Broccoli | 0 |
| Asparagus lettuce | 0 | Fried chicken chop | 7.3 | Braised beef | 13.7 | Sweet and Sour Chicken | 57.4 |
| Soup | 14.8 | Soup | 8.7 | Soup | 6 | Soup | 6 |
| Rice | 6 | Rice | 6 | Rice | 6 | Rice | 6 |
| Braised eggplant | 0 | Squash | 0 | Dry fried bean | 0 | Braised meatballs | 0 |
| Fried Chicken Chop | 45.5 | Flowering cabbage | 13.6 | Deep fried chicken legs | 11.7 | Green pepper, potato and eggplant | 6.9 |
| Flowering cabbage | 0 | Braised beef | 7.8 | Chinese cabbage | 0 | cabbage | 17.1 |
| Soup | 16.2 | Soup | 11 | Soup | 7.5 | Soup | 6.4 |
| Biscuit | 14.4 | Biscuit | 14.4 | Biscuit | 14.4 | Biscuit | 14.4 |
| Pear | 0 | Pear | 0 | Apple | 0 | Pear | 0 |
| Period 2 | | | | | | |  |
| Oyster sauce fried lettuce | 10.3 | Chinese kale | 0 | Shredded potatoes | 11.9 | Squash | 0 |
| Bean paste bun | 0 | Fried cake | 14.4 | Steamed roll | 0 | Bean paste bun | 0 |
| soymilk | 0 | Porridge | 0 | Soymilk | 0 | Porridge | 0 |
| Mustard tuber | 25.5 | Mustard tuber | 25.5 | Mustard tuber | 25.5 | Mustard tuber | 25.5 |
| Egg | 228.6 | Egg | 228.6 | Egg | 228.6 | Egg | 228.6 |
| Rice | 6 | Rice | 6 | Rice | 6 | Rice | 6 |
| Squash | 0 | Dry fried cauliflower | 0 | Squash | 0 | Braised beef | 13.7 |
| Chinese kale | 0 | Shredded potatoes | 17.1 | Cabbage | 6.6 | Braised eggplant | 0 |
| Stewed chicken wings | 6.1 | Braised pork | 18.9 | Fried chicken chop | 26.9 | Green pepper | 10.1 |
| Soup | 6.4 | Soup | 6 | Soup | 6 | Soup | 6 |
| Rice | 6 | Rice | 6 | Rice | 6 | Rice | 6 |
| Braised beef | 87.9 | Cabbage | 6.6 | Braised meatballs | 0 | Dry fried bean | 8.6 |
| Braised eggplant | 0 | Deep fried chicken legs | 11.7 | Green pepper, potato and eggplant | 9 | Fried chicken chop | 26.6 |
| Pakchoi | 0 | Parsley fried carrot | 6.4 | Flowering cabbage | 0 | Broccoli | 16.8 |
| Soup | 9.3 | Soup | 6 | Soup | 9.2 | Soup | 9.4 |
| Biscuit | 14.4 | Biscuit | 14.4 | Biscuit | 14.4 | Biscuit | 14.4 |
| Apple | 0 | Pear | 0 | Apple | 0 | Banana | 20.7 |
| Period 3 | | | | | | |  |
| Oyster sauce fried lettuce | 0 | Chinese kale | 30.1 | Shredded potatoes | 17.1 | Parsley fried carrot | 6.4 |
| Bean paste bun | 17.8 | Fried cake | 0 | Steamed roll | 0 | Bean paste bun | 17.8 |
| Soymilk | 7.8 | Porridge | 0 | Soymilk | 7.8 | Porridge | 0 |
| Mustard tuber | 25.5 | Mustard tuber | 25.5 | Mustard tuber | 25.5 | Mustard tuber | 25.5 |
| Milk | 269 | Milk | 269 | Milk | 269 | Milk | 269 |
| Rice | 6 | Rice | 6 | Rice | 6 | Rice | 6 |
| Shredded potatoes | 0 | Braised pork | 7 | Braised sparerib | 0 | Braised beef | 13.7 |
| Flowering cabbage | 0 | Flowering cabbage | 12 | Squash | 0 | Braised eggplant | 0 |
| Sweet and Sour Chicken | 57.4 | Shredded potatoes | 17.1 | Cabbage | 17.1 | Green pepper | 6.6 |
| Soup | 11.9 | Soup | 9.4 | Soup | 10.6 | Soup | 11.9 |
| Rice | 6 | Rice | 6 | Rice | 6 | Rice | 6 |
| Braised beef | 7.7 | Parsley fried carrot | 6.4 | Braised meatballs | 0 | Dry fried bean | 6.6 |
| Pakchoi | 0 | Deep fried chicken legs | 19.3 | Green pepper, potato and eggplant | 0 | Fried chicken chop | 0 |
| Braised eggplant | 0 | Cabbage | 6.6 | Flowering cabbage | 13 | Broccoli | 13.6 |
| Soup | 6 | Soup | 9.4 | Soup | 11.9 | Soup | 9.4 |
| Biscuit | 14.4 | Biscuit | 14.4 | Biscuit | 14.4 | Biscuit | 14.4 |
| Apple | 0 | Pear | 0 | Pear | 0 | Banana | 20.7 |
